# The use of a convolutional neural network to automate radiologic scoring of computed tomography of paranasal sinuses

**DOI:** 10.1186/s12938-025-01376-7

**Published:** 2025-04-27

**Authors:** Daniel J. Lee, Mohammad Hamghalam, Lily Wang, Hui-Ming Lin, Errol Colak, Muhammad Mamdani, Amber L. Simpson, John M. Lee

**Affiliations:** 1 Department of Otolaryngology-Head and Neck Surgery, Unity Health TorontoSt. Michael’s Hospital, University of Toronto, 30 Bond Street, 8 Cardinal Carter Wing, Toronto, ON M5B 1W8 Canada; 2 Division of Otolaryngology-Head and Neck Surgery, Department of Surgery, North York General Hospital, University of Toronto, Toronto, Canada; 3 School of Computing, Queen’s University, Kingston, ON Canada; 4 Department of Electrical Engineering, Qazvin Branch, Islamic Azad University, Qazvin, Iran; 5 The Li Ka Shing Centre for Healthcare Analytics Research & Training, Unity Health Toronto, St. Michael’s Hospital, Toronto, Canada; 6 Department of Medical Imaging, Unity Health Toronto, University of Toronto, Toronto, Canada

**Keywords:** Artificial intelligence, Machine learning, Rhinology, Chronic rhinosinusitis

## Abstract

**Background:**

Chronic rhinosinusitis (CRS) is diagnosed with symptoms and objective endoscopy or computed tomography (CT). The Lund–Mackay score (LMS) is often used to determine the radiologic severity of CRS and make clinical decisions. This proof-of-concept study aimed to develop an automated algorithm combining a convolutional neural network (CNN) for sinus segmentation with post-processing to compute LMS directly from CT scans.

**Results:**

Radiology Information System was queried for outpatient paranasal sinus CTs at a tertiary institution. We identified 1,399 CT scans which were manually labelled with LMS of individual sinuses. Seventy-seven CT scans with 13,668 coronal images were segmented manually for individual sinuses. Our model for segmentation achieved a mean Dice score of 0.85 for all sinus regions, except for the osteomeatal complex. For individual Dice scores were 0.95, 0.71, 0.78, 0.93, 0.86 for the maxillary, anterior ethmoid, posterior ethmoid, sphenoid, and frontal sinuses, respectively. LMS was computed automatically by applying adaptive image thresholding and pixel counting to the CNN’s segmented regions. A convolutional neural network (CNN) model was trained to segment each sinus region. Overall, the LMS model showed a high degree of accuracy with a score of 0.92, 0.99, 0.99, 0.97, 0.99, 0.86 for the maxillary, anterior ethmoid, posterior ethmoid, sphenoid, and frontal sinuses, respectively.

**Conclusions:**

Reporting of paranasal sinus CT can be automated and potentially standardized with a CNN model to provide accurate Lund–Mackay score.

## Background

Chronic rhinosinusitis (CRS) is a prevalent inflammatory disease of the paranasal sinuses, which impacts 5–12% of general population [1]. CRS has a significant impact on quality of life comparable to or worse than those with angina, chronic heart failure or asthma [1–4]. In addition, patients with CRS have substantial healthcare costs between $10 and $13 billion dollars a year as well as decreased work productivity [1, 5]. With the impact of CRS on patients and healthcare system, it is paramount to have accurate diagnosis for the optimal management of CRS. The diagnosis is made based on the presence of symptoms (nasal obstruction/congestion, facial pain, nasal discharge or reduction or loss of smell) in addition to objective findings on nasal endoscopy or radiological imaging, such as computer tomography (CT) [1]. In fact, paranasal sinus CT is widely ordered by otolaryngologists and non-otolaryngologists to assess the presence, extent, and severity of CRS.

Despite the importance of the paranasal sinus CT for objectively diagnosing and quantifying sinus disease, little progress has been made to standardize and optimize their interpretation. The most common and validated measure of sinus disease opacification is the Lund–Mackay score (LMS), which is used extensively both in clinical and research settings [1, 6]. However, this is rarely reported on standard CT reports, which may be partly due to the time consuming nature of calculating this score. In fact, many otolaryngologists in Canada reported dissatisfaction with the reporting of CT scans [7]. Many otolaryngologists in practice may not have access to neuroradiologists and feel that there is a need to improve radiology reporting for more clinically relevant information [7]. Even between two academic centres, there was a large discrepancy in the quality of radiology reporting in this study [7]. There likely is a larger variability among all the healthcare facilities in Canada. In addition, in clinical practice, access to the images may not be immediately available, which highlights the importance of a CT report that conveys an objective measure of sinus disease. Therefore, there is a need to standardize the reporting of CT scans of sinus to improve the quality of diagnosis and management of CRS by incorporating standardized scores, such as LMS.

Recently, convolutional neural networks (CNNs) have gained prominence in medical image analysis due to their ability to automatically learn hierarchical features, such as edges, shapes, and textures, directly from raw imaging data [8]. In medical imaging, the validation of machine learning models involves rigorous evaluation processes, including internal and external validation, to ensure generalizability and reliability across diverse populations and imaging modalities. These steps are critical for translating such algorithms into clinical practice, where they must perform robustly and accurately in real-world scenarios. The application of CNNs to paranasal sinus CT scans is particularly promising, given the anatomic complexity of the sinuses. These structures are highly variable in size, shape, and pneumatization across individuals, which poses a significant challenge for automated analysis. Previously, machine learning has been previously employed to quantify the percentage of sinus opacification and has been shown to correlate with clinical improvement following treatment without reporting of automated LMS [9–11]. While LMS has its inherent drawback with the coarse structure, LMS has been shown to be simple to perform with high interobserver reliability and, therefore, has been widely used in clinical and research settings [1, 6]. However, manual scoring of LMS is time-intensive and requires specialized knowledge, making it inaccessible to non-experts and less feasible for large data sets. Automating this process using CNNs can overcome these barriers by efficiently segmenting individual sinuses and providing objective, quantitative interpretations of sinus opacification. In regions where otolaryngologists or radiologists with sinus expertise are scarce, CNN-based tools could offer accurate and reliable evaluations, bridging the gap in care and improving diagnostic workflows in underserved areas. However, the use of artificial intelligence (AI) in medical diagnosis carries ethical implications, including concerns about algorithmic bias, accountability, and transparency. To address these challenges, it is essential to utilize comprehensive and diverse radiology databases that ensure equitable representation of various patient populations. This approach not only helps minimize bias but also promotes fairness and enhances the generalizability of AI models in real-world clinical settings. Many previous models were developed using data sets that likely had selection bias, as they predominantly consisted of patients with chronic rhinosinusitis or those undergoing evaluation for chronic rhinosinusitis [9–11]. This limited representation could affect the model’s ability to perform effectively across a broader range of clinical scenarios.

The goal of this study is to develop and validate a proof-of-concept CNN-based algorithm to automate LMS scoring and segmenting individual sinuses from paranasal CT scans using a radiology database with consecutive images to minimize selection bias. Specifically, the CNN will be used to first segment the paranasal sinuses. Then, the LMS will be computed directly from these segmented regions using adaptive thresholding and pixel counting. This model aims to provide accurate, accessible, and objective interpretation of sinus CT scans, thereby addressing the challenges posed by anatomic complexity and improving clinical workflows in diverse healthcare settings.

## Results

This study serves as a proof-of-concept for the development of an automated system to evaluate sinus opacification using the LMS and sinus segmentation model.

### Sinus segmentation results

**Fig. 1:**
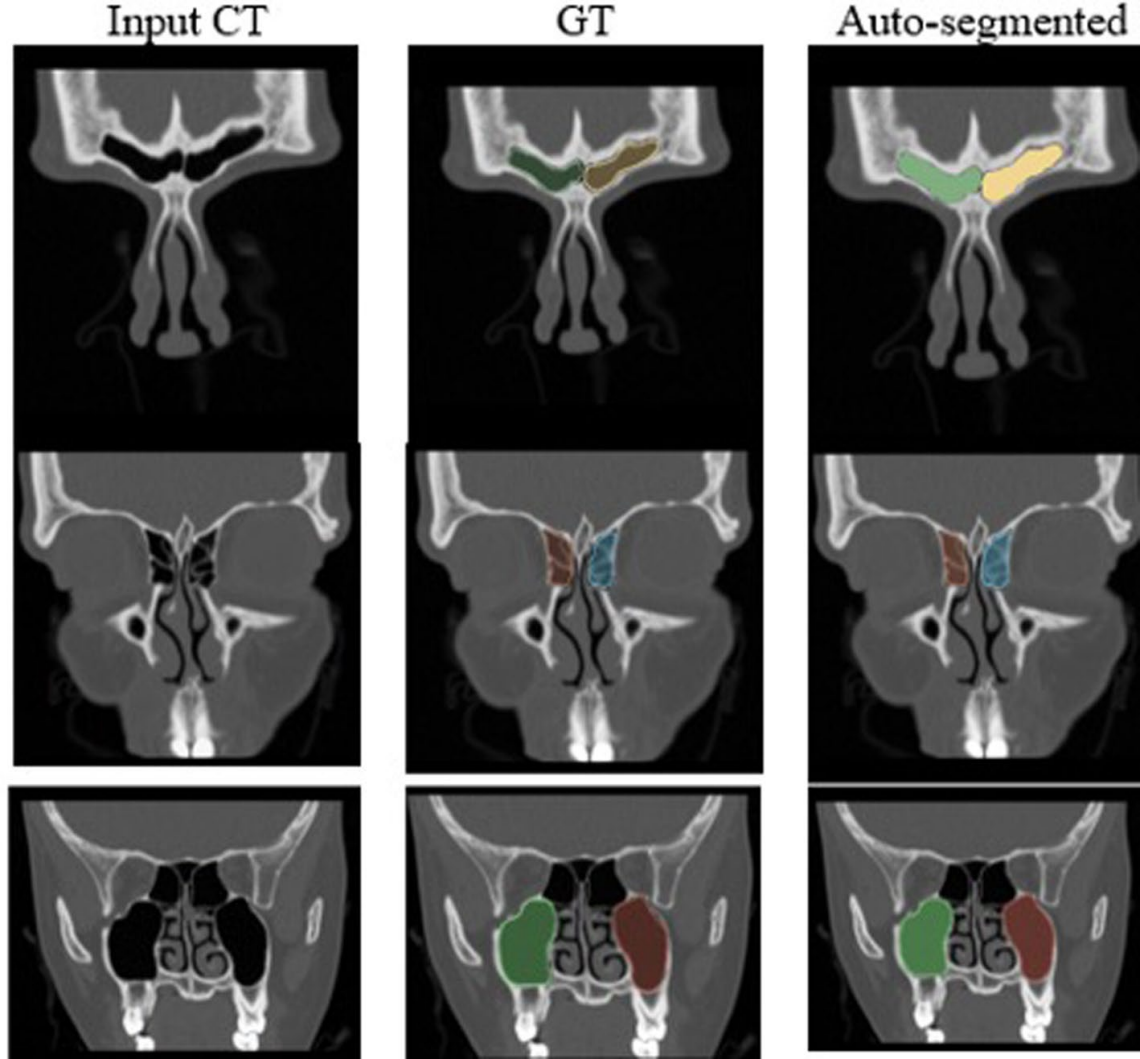
Examples of segmentation results. From top to bottom, frontal, ethmoid, and maxillary, sinus. *GT* ground truth

Table 1 presents the results of the segmentation model for different sinus regions using fivefold cross-validation. Figure 1 demonstrates an example of input, ground truth and auto-segmentation. The model achieved a mean Dice score of 0.85 for all sinus regions except for OMC. Specifically, the model produced the following Dice scores: 0.95 for the maxillary sinus, 0.71 for the anterior ethmoid sinus, 0.78 for the posterior ethmoid sinus, 0.93 for the sphenoid sinus, and 0.86 for the frontal sinus. These results demonstrate a high degree of accuracy in segmenting the paranasal sinuses, with the exception of the OMC, which had a notably lower Dice score of 0.18.

**Table 1:** Average dice scores for segmentation of sinus regions

	Frontal	Anterior ethmoid	posterior ethmoid	Maxillary	Sphenoid	OMC
DSC score	0.86	0.71	0.78	0.95	0.93	0.18
95% CI^a^	0.79–0.92	0.64–0.78	0.67–0.88	0.94–0.97	0.93–0.96	0.07–0.29

^a^*CI* confidence interval

Importantly, the use of fivefold cross-validation indicates that there is high consistency in the model's performance across different folds. This suggests that the model is robust and performs reliably across multiple subsets of the data, providing confidence in its generalizability.

### LMS model results

Table 2 presents the accuracy, weighted precision, weighted recall, and weighted F1 score of the LMS model for different sinus regions in the cohort of patients. The model achieved high accuracy values across all sinus regions, with the accuracy exceeding 0.9 in all regions except for the Ostiomeatal Complex (OMC). Similar trends were observed for weighted precision, recall, and F1 scores. The results indicate that the model performs reliably across most sinus regions, demonstrating high consistency in its predictions.

**Table 2:** Average accuracy value for LMS model

	Frontal	Anterior ethmoid	Posterior ethmoid	Maxillary	Sphenoid	OMC
Accuracy	0.99	0.99	0.99	0.92	0.97	0.71
Weighted precision	0.99	0.99	0.99	0.93	0.97	0.81
Weighted Recall	0.99	0.99	0.99	0.91	0.96	0.71
Weighted F1 score	0.99	0.99	0.99	0.92	0.97	0.62

### Performance comparison

The LMS model results indicated high accuracy, weighted precision, recall, and F1 scores across all sinus regions, with the exception of the OMC. These results underscore the model's potential in providing accurate automated evaluations of sinus opacification. However, it is important to note that the OMC region exhibited lower performance, highlighting the need for further refinement in this specific region.

The segmentation model demonstrated high consistency in segmentation performance across various sinus regions. With a mean Dice score of 0.85, the model performed particularly well in segmenting the maxillary, sphenoid, and frontal sinuses. However, the significantly lower performance in the OMC region suggests that additional training and improvements may be necessary to handle complex anatomical structures more effectively.

## Discussion

In this study, we trained and evaluated a proof-of-concept CNN method for automated assessment of paranasal sinus CT segmentation and scoring system using LMS in a large set of data. We demonstrate that a CNN model can automatically segment and provide LMS per each sinus region with a high degree of accuracy in all sinuses with the exception of OMC. As manual scoring is time-consuming and relies on clinical expertise and interpretation, this CNN method can be an attractive model to be further refined and implemented in a large system for automated scoring of paranasal sinus CT scans and can be used to help standardize CT sinus reporting across institutions.

Although the overall sinus segmentation model had a high Dice score of 0.85, the accuracy was not as high with the ethmoid sinuses and OMC. This discrepancy among different regions of paranasal sinuses likely derives from the availability of well-defined anatomic boundaries. While frontal, maxillary, and sphenoid sinuses have defined bony boundaries, ethmoid sinuses and the OMC may not have these borders particularly in patients who had endoscopic sinus surgery in the past. The OMC, in particular, may also have variable anatomy depending on the status of previous surgery, which we hope to incorporate in our future model. Specifically, the OMC will likely need to be defined in models without previous surgery, to optimize deep learning and increase the Dice score. Our future model will incorporate the presence of previous surgery and increase sample size to improve the accuracy of the OMC and ethmoid sinus. Deviations from standard sinus anatomy, such as supraorbital ethmoid or spheno-ethmoid (Onodi) air cells, can also contribute to lower Dice scores, given their low prevalence in the data set. Despite the shortcomings with sinus segmentation, the LMS model demonstrates great accuracy, precision, recall and F1 scores with a value of over 0.90 in all sinuses. This demonstrates robust and superior performance of the LMS model in all the measures of a ML model and its potential applicability in clinical practice.

There have been other work in the use of deep learning techniques to help quantify the paranasal sinus opacification [9, 10, 12]. These publications demonstrate the development and use of CNN model for volumetric analysis of paranasal sinus opacification. The model has been shown to be very effective in the evaluation of therapies in clinical studies that use paranasal sinus CT before and after therapeutics. However, while this model presents a percentage opacification in the overall sinuses, it does not generate the LMS. While the LMS does have criticism for having its coarse scale with respect to the score of “1,” it still remains the gold standard which is used clinically and in research settings (such as clinical trials) as a marker of sinus disease severity. In addition, the LMS has a number of strengths, such as the ease of use, high interobserver reliability and correlations with other markers of disease severity [6, 13]. In fact, while LMS of 2 or less has an excellent negative predictive value, LMS of 5 or greater has a great positive predictive value to indicate the presence of CRS [1, 14, 15]. Finally, distinct from previous studies, to minimize potential selection bias arising from using a select cohort of patients, we used consecutive images from a large radiology database. This has implication in the ethics of application of AI in healthcare in reducing systemic, algorithmic bias and ensuring fairness. In our future studies beyond the proof-of-concept model, we hope to incorporate and compare clinical variables from our clinical patients. The ultimate goal of this model is to standardize the reporting of LMS across multiple hospitals within a region. This can be particularly helpful for primary care physicians or other non-otolaryngologists who order CT sinus scans, as the report is standardized with LMS, which non-otolaryngologists can use as referring information. In turn, otolaryngologists can use this to appropriately triage the patients. For radiologists, this can be a simple, non-time consuming augmentation to the report. By incorporating LMS, we aim to facilitate more consistent and meaningful comparisons of sinus disease across healthcare settings, improving the reliability and generalizability of clinical assessments.

This study is not without limitations. This study used a retrospective, historic data set based on a single tertiary hospital, which may limit the generalizability and external validity with uniform protocols. Ideally, the model would be evaluated as part of a prospective controlled trial at multiple external institutions. Training the model with data from additional institutions could help with model robustness as a result of including different CT scanners, imaging protocols, and patient population in the training data set. There are lower performance markers in our studies particularly with respect to OMC and ethmoids. We hope to increase the performance levels by addressing whether surgery has been performed or not in our data set. In addition, this will be compared with a historic clinical data set to further augment our model. In addition, future work should investigate how the model performs across different clinical settings, including hospitals without direct access to otolaryngologists or specialized radiologists. This would enhance the accessibility of automated sinus evaluation in underserved areas. Furthermore, there may be errors related to manual data entry which are expected to be insignificant with the volume of scans analyzed, but are nevertheless a consideration for model inaccuracy. We acknowledge that this study did not contain any clinical data as our aim was to develop a pure machine learning model based on consecutive radiology images to reduce any potential systemic, algorithmic, and selection biases. In the future, we aim to further refine the model, integrate this into clinical practice and validate it in clinical settings. We acknowledge the less than ideal Dice scores in ethmoids and OMC. In the future, we are intending to further improve our model by incorporating the history of previous surgery in our model. We acknowledge that many recent papers incorporate percentage opacification. While it is yet to be clear whether the exact percentage opacification of each sinus will possess clinical importance, we will incorporate percentage opacification in our future model, as we further develop and refine our model in a multi-institutional fashion. Although performance metrics such as accuracy, precision, recall, and F1 score were reported, this study did not include confidence intervals, which are essential for understanding the precision and reliability of the model’s predictions. Future studies will benefit from incorporating confidence intervals to assess the significance of performance differences across sinus regions and refine the model’s robustness. The absence of confusion matrices limits our ability to fully understand the types of errors the model makes. Future work should include confusion matrices to explore misclassifications in greater detail. This will help identify whether errors are random or systematic, and provide insights into potential areas of improvement, particularly in challenging regions, such as the OMC. In addition, future work can involve investigation into the use of alternative methods, such as Swin-UNet [16], DeepLabV3 + [17], and multi-scale feature fusion (MSFF) [18, 19].As the current study focused on the development of the proof-of-concept model, inter-observer agreement between multiple experts was not assessed. For future studies, evaluating the model's performance against expert annotations and comparing it to inter-observer agreement will provide a clearer benchmark for the model's reliability and clinical utility.

Regardless, our proof-of-concept models (both scoring the LMS and contouring of the sinuses) have the potential to standardize CT sinus reporting across institutions in a clinically meaningful way and provide an objective measure of sinus opacification. This can be applied in a variety of clinical settings, addressing barriers in loading CT images across different public and private imaging centres and saving significant time per each outpatient encounter spent otherwise calculating the score. This can ultimately help with clinical decision making in helping both confirm the diagnosis of chronic rhinosinusitis while also assessing the severity of sinus disease.

## Conclusion

Our proof-of-concept CNN model demonstrates great accuracy in automatically scoring and segmenting individual paranasal sinuses on CT scans. With this model, there is a potential to help standardize CT sinus reporting in an objective and clinically meaningful way.

## Methods

This study (LKS-CHART REB#16-371) was approved by the Research Ethics Board. Due to retrospective nature of this study, informed consent was waived and approved by the Research Ethics Review Board.

### Data collection and image selection

The radiology information system was queried for outpatient non-contrast paranasal sinus CT scans conducted at a tertiary academic institution using Nuance mPower. The selected scans were exported from the Picture Archiving and Communication System (PACS) and anonymized using RSNA Anonymizer to remove all identifying patient information. The data was stored in a secure, password-protected hospital network. The imaging protocol for the CT scans was consistent, employing coronal bone algorithm with slice thickness of 2 mm, suitable for evaluating paranasal sinuses. Scans with imaging artifacts, motion blur, or incomplete data sets were excluded. Sinonasal imaging with neoplastic processes or trauma features was also excluded to standardize the data set. To provide a proof-of-concept, 1399 consecutive CT scans were chosen for LMS labeling based on sample size feasibility, data availability, and institutional resources. Of these, 77 representative scans (13,668 coronal slices) were selected for sinus segmentation to balance computational feasibility and data diversity. Representative scans were chosen to include varying degrees of opacification and anatomical diversity, ensuring the model's robustness across different clinical presentations.

### Preprocessing and annotation

Images were preprocessed using normalization techniques to standardize pixel intensity values, resizing to a 448 × 512 resolution, and cropping to focus on regions of interest (paranasal sinuses). No augmentation techniques (e.g., rotation and flipping) were applied, as the data set was deemed sufficient in diversity. Image quality variations, including minor motion artifacts, were managed using expert review to either exclude affected images or annotate around imperfections.

Manual annotations and segmentations were performed using md.ai, a secure, web-based annotation platform (https://www.md.ai), which supports collaborative annotation for medical imaging studies [20]. Each sinus region (right and left frontal, anterior ethmoid, posterior ethmoid, maxillary, sphenoid, and osteomeatal complex [OMC]) was manually segmented and labeled by a trained team of otolaryngologists and neuroradiologists. A detailed sinus segmentation and LMS scoring manual was developed and approved by expert rhinologists and a radiologist (DJL, JL, and EC).

Disagreements in labeling were resolved through consensus meetings, where a majority agreement or arbitration by a senior rhinologist was used to finalize annotations.

### Deep learning algorithm development and model architecture

#### Sinus segmentation model

A 2D U-Net architecture, specifically based on the 2D implementation of the nnUNet framework [21], was chosen for sinus segmentation due to its proven effectiveness in medical image analysis. The 2D U-Net was selected over other approaches after empirical testing showed superior performance for our specific task. Compared to a 3D configuration, the 2D U-Net achieved a Dice Similarity Coefficient (DSC) 3 percentage points higher, making it more suitable for sinus segmentation. We attribute this improved performance to the following factors: 1) limited availability of annotated volumetric data, which is required for effective 3D modeling, and 2) the slice-localized nature of sinus structures in CT scans, where inter-slice context is less critical for segmentation. For similar reasons, 3D multi-feature attention-based pruning (MFA) [22], and 3D mobile residual U-Net (MRU-Net) [23, 24] were considered but not used for this proof-of-concept study.

The patch size of 448 × 512 was determined based on the median coronal plane size of the images in our database, after cropping the regions of interest and resampling. This ensures that the model captures the full range of variation in the data. All convolutions use a kernel size of 3 × 3 with a LeakyReLU activation function. The U-Net encoder, based on the 2D nnUNet architecture, downsamples the feature map size from 448 × 512 (input patch) to 480 × 7 × 4 (bottleneck layer) through six convolution layers with a stride of 2. The decoder upsamples the feature maps using 2D transposed convolutions, incorporating skip connections between corresponding encoder and decoder layers to preserve spatial information.

While alternative architectures such as Swin-UNet [16], DeepLabV3 + [17], and multi-scale feature fusion (MSFF) [18, 19] could be considered, they require significantly larger data sets or are designed for natural image segmentation, which introduces challenges in anatomical accuracy for our task and potentially require more memory and computation than the 2D U-Net model. Given the limited labeled data, the need for precise spatial information and computational resource restraint as a proof-of-concept model, the 2D U-Net provided a better balance between computational efficiency and segmentation accuracy.

We trained and validated our model using 77 subjects to minimize cross entropy and Dice loss through fivefold validation. Our implementation is developed employing PyTorch on an NVIDIA A100 GPU with 40 GB of RAM. Adam optimizer (learning rate of 0.01, weight decay rates of β₁ = 0.9 and β₂ = 0.999) was used to update model weights during training. The model was trained using a batch size of 16 images per iteration for 100 epochs, with early stopping applied to prevent overfitting based on validation loss. Dropout layers were incorporated to further reduce overfitting and improve generalization. Dice loss and cross-entropy loss were utilized as the primary loss functions to optimize segmentation accuracy, while fivefold cross-validation was conducted to ensure robustness and evaluate model performance across different subsets of the data.

#### LMS model development

**Fig. 2:**
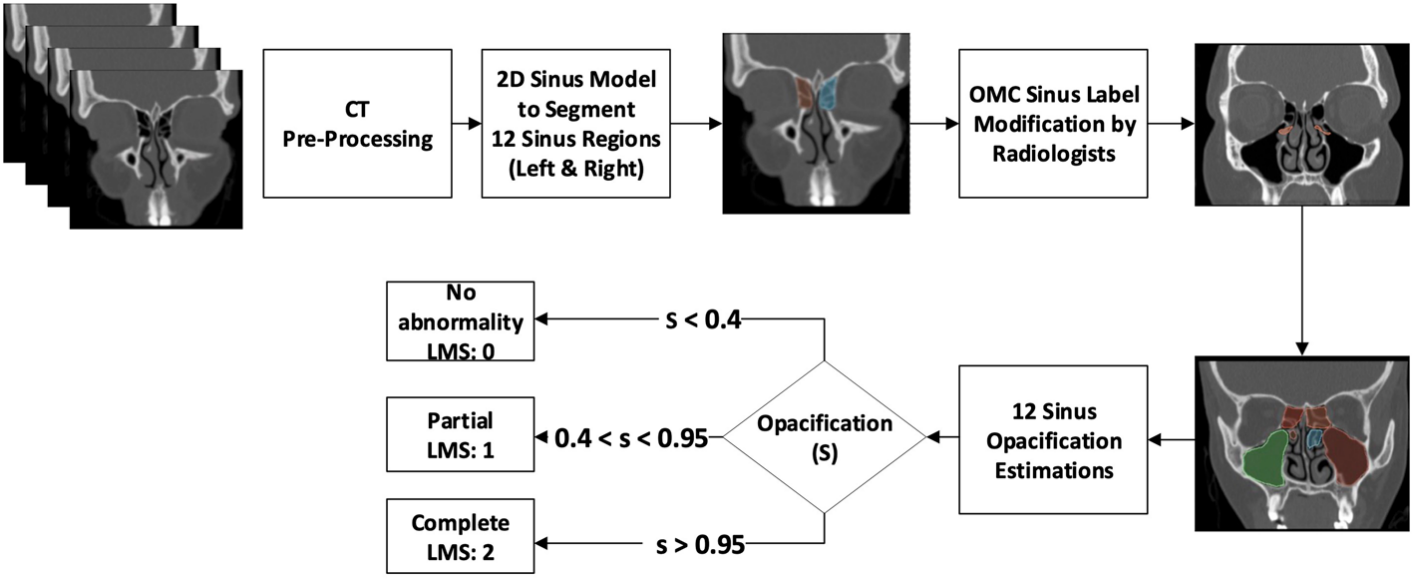
Architecture of the ML model

An overview of the ML model is presented in Fig. 2. The LMS model was designed to quantify sinus opacification using an image thresholding approach combined with pixel counting. Thresholding separated opacified regions from surrounding tissues, with pixel counts corresponding to the degree of opacification. By determining an appropriate threshold value based on empirical observations and statistical analysis, the opacified regions were segmented. After obtaining the segmented opacified regions, the number of pixels within each region was counted. This pixel count served as a quantitative measure of the degree of opacification, representing the extent of sinus area inflammation involvement. A higher pixel count indicated a more severe degree of opacification, signifying a more severe sinus disease. LMS scoring was automated as follows:

- Score 0: opacification < 0.01
- Score 1: opacification between 0.01 and 0.95
- Score 2: opacification > 0.95

Validation involved comparing automated scores with expert manual LMS assessments across the 1400 labeled CT scans.

#### Performance metrics

The performance of the segmentation and LMS models was evaluated using several key metrics. Accuracy was measured by the proportion of correctly classified sinus regions. Precision and recall were used to evaluate the model's ability to minimize false positives and false negatives, respectively. The F1 score, which is the harmonic mean of precision and recall, provided a balanced measure of the model's performance in both detecting and avoiding errors. The Dice score is calculated by dividing the overlap between ground truth (GT) and predicted images by half the total number of pixels in both images. It is used as a statistical validation metric to evaluate the performance of both the reproducibility of GT segmentations and the spatial overlap accuracy of automated probabilistic fractional segmentation of CT images. There is a range of Dice values between 0 and 1, with 1 denoting the highest accuracy without any overlapping errors and 0 indicating the lowest accuracy.

## Data Availability

No datasets were generated or analysed during the current study.
